# Psoas Abscess and Pott’s Disease Masked by Concomitant Invasive Staphylococcus aureus Disease: A Case of Misleading Diagnosis

**DOI:** 10.7759/cureus.47679

**Published:** 2023-10-25

**Authors:** Patricia Bernardo, Rita Gonçalves Pereira, Carla Nobre, Filipa Silva, Vanessa Figueiredo

**Affiliations:** 1 General Surgery, Centro Hospitalar Barreiro-Montijo, Barreiro, PRT; 2 General Surgery, Centro Hospitalar de Setubal, Setúbal, PRT; 3 Intensive Care Unit, Centro Hospitalar de Setúbal, Setúbal, PRT; 4 Intensive Care Unit, Centro Hospitalar de Setubal, Setubal, PRT

**Keywords:** adult intensive care, general surgery, coinfection, spinal tuberculosis, methicillin-susceptible staphylococcus aureus infection, psoas abscess

## Abstract

Psoas abscess is a rare infection classified as primary or secondary depending on the etiology of infection. *Staphylococcus aureus* is considered the most frequent causative agent. Nevertheless, psoas abscess persistent lack of improvement or any relapse after successful treatment should remind us to exclude other potential diagnoses. Although less frequently, Pott’s disease is still one of the predisposing causes, especially in patients with immunocompromised status. This clinical condition has an indolent course and requires a high index of suspicion to avoid severe morbidity. Early recognition and targeted treatment are the principal means of ensuring tuberculosis control. Here we report a very interesting case of a psoas abscess and Pott’s disease in a patient suffering from a misleading diagnosis of invasive staphylococcal disease.

## Introduction

Psoas (or iliopsoas) abscess is a collection of pus in an extraperitoneal space that contains the iliopsoas muscle. This rare infection was first described by Mynter [1] in 1881 and it can be classified as primary or secondary depending on the etiology of infection. Primary psoas abscess is presumably a result of haematogenous or lymphatic spread from an occult infectious process. Secondary abscess arises via contiguous dissemination from an adjacent infectious focus, such as vertebral bodies and discs, gastrointestinal tract, hip joints and other sites [2].

Despite the decreasing incidence of tuberculosis in developed countries, Pott's disease is still one of the predisposing causes of psoas abscess development. It is the most alarming form of musculoskeletal tuberculosis and its indolent clinical course requires a high index of suspicion to avoid severe morbidity, such as irreversible neurologic complications, including plegia, paresis, impaired sensation or nerve root pain [3].

Here we report a very interesting case of a psoas abscess and Pott’s disease in a patient suffering from a misleading diagnosis of invasive staphylococcal disease. Simultaneous infections in tuberculosis are rare and this particular coinfection has not been reported to date.

## Case presentation

**Figure 1:**
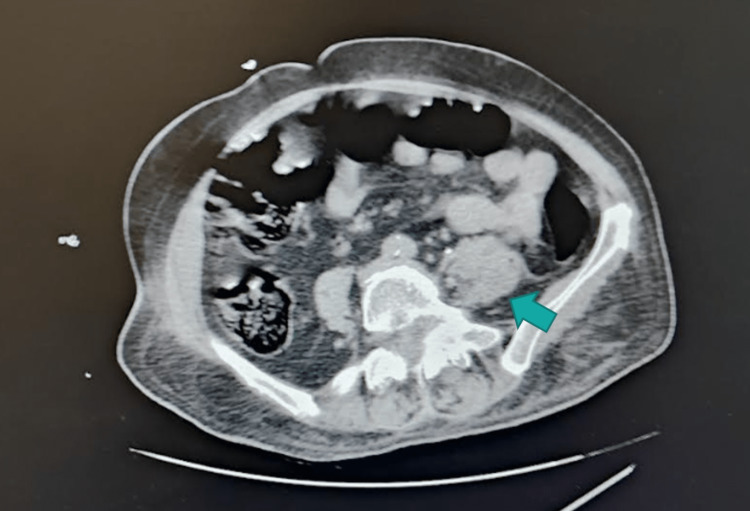
Left psoas muscle Computed axial tomography scan of the abdomen demonstrating an intramuscular collection measuring about 10 centimeters in the left psoas muscle (arrow).

A 72-year-old Caucasian woman, autonomous presented to the emergency room with fever and altered mental status. The only relevant personal history is non-insulin-dependent diabetes mellitus. On admission, her body temperature was 38.3ºC, respiratory rate 25/min, heart rate 150 beats/min and blood pressure 65/48 mmHg. Remaining physical examination was unremarkable. Laboratory findings showed white cell count 14.8×109 L (4 to 11×109 L) and C-reactive protein 28.2 mg/dL (<0.5 mg/dL). In order to clarify the source of infection, computed tomography (CT) scans were performed. Head and chest CT scans were negative, while CT of the abdomen and pelvis revealed an intramuscular collection measuring about 10cm in the left psoas extending into the left iliac fossa, without other relevant findings (Figure 1).

Thus, empirical antimicrobial therapy was initiated with piperacillin/tazobactam 4g/0.5g every eight hours. The patient's daughter provided consent for surgical management and the abscess was drained via open retroperitoneal approach. After the surgical procedure, the patient was admitted to the Intensive Care Unit (ICU). As a consequence of persistent deteriorated mental status and septic shock, a lumbar puncture was performed and antibiotic therapy was adjusted to vancomycin, ceftriaxone and ampicillin, as cerebrospinal fluid (CSF) biochemical and cytological analysis was compatible with acute bacterial meningitis.

A few days later, blood cultures, pus from psoas abscess and CSF yielded gram-positive methicillin-susceptible *Staphylococcus aureus* (MSSA). Based on confirmed invasive MSSA infection, the patient underwent transesophageal cardiac ultrasound which revealed a vegetation of the anterior mitral leaflet measuring 10x8mm, without significant functional alterations. Therefore, antibiotic was changed to high-dose flucloxacillin 2g every six hours, according to antibiotic sensitivity tests.

Despite all the appropriate approaches, the patient did not improve remarkably and psoas abscess recurred one week after the drainage. Facing the new findings, the team decided to reoperate through the same surgical wound and the surgery was carried out uneventfully. New pus samples were collected and sent for bacteriological analysis.

**Figure 2:**
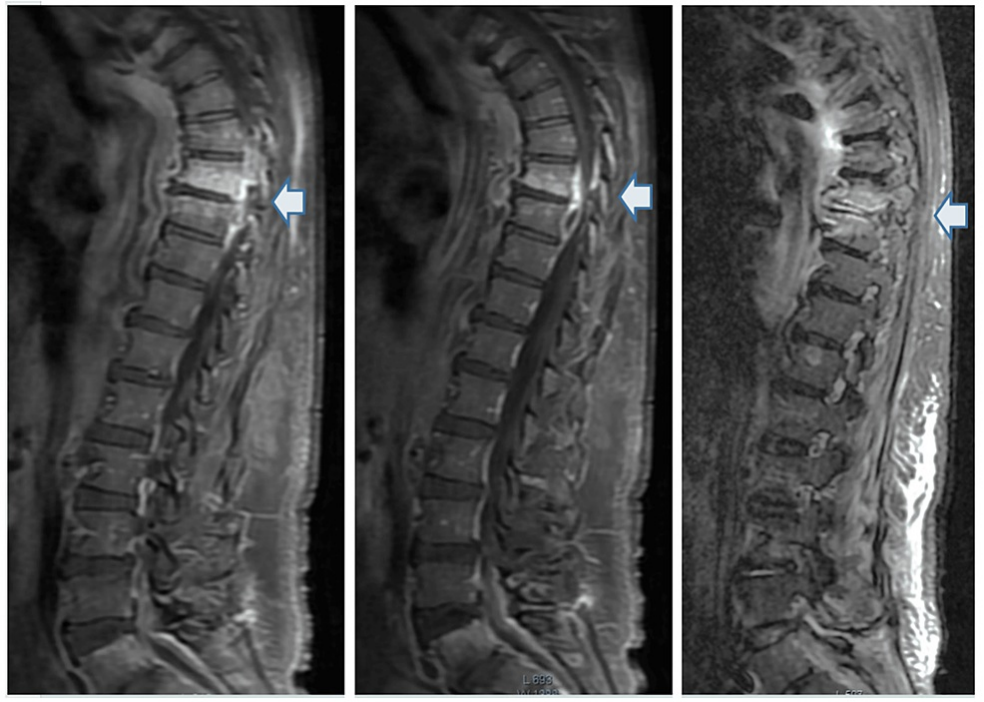
Pott's disease Magnetic resonance imaging (MRI) of the spine (sagittal view) showing exuberant dorsolumbar spondylodiscitis and epidural empyema with mass effect on the spinal cord (arrows).

Due to abscess recurrence and new onset of muscle strength deficits in the lower limbs, further investigations were conducted for accurate diagnosis. Magnetic resonance imaging (MRI) of the spine showed exuberant dorsolumbar spondylodiscitis and epidural empyema with mass effect on the spinal cord (Figure 2).

Unexpectedly, one month after the initial diagnosis, the results of the pus culture came back positive for *Mycobacterium tuberculosis* (MTB) and a four-drug regimen (isoniazid, rifampin, pyrazinamide and ethambutol) was associated to the ongoing antibiotic therapy.

After four weeks of targeted therapy for MTB and MSSA, it was possible to obtain satisfactory clinical and imagiological results and the patient was discharged. At three-month follow-up, the patient was still alive, recovering with rehabilitation of paraparesia caused by the spinal cord compression, a complication of the late diagnosis of spinal tuberculosis.

## Discussion

Staphylococcal disease is an increasing global concern with a higher complication rate of septic shock than any other bacteria [3,4]. Given the diagnosis of psoas abscess, *Staphylococcus aureus* is considered the most frequent causative agent [2]. Accordingly, one would expect the MSSA invasive disease to be the clinical problem of this patient.

Spine tuberculosis, also called Pott's disease, accounts for almost half of all cases of musculoskeletal tuberculosis [5]. Rarely, psoas abscess can be related to Pott's disease and it is a diagnostic challenge because of its indolent clinical course [5,6].

Psoas abscess persistent lack of improvement or any relapse after successful treatment should remind us to exclude other potential diagnoses. Patients with known risk factors, like diabetes and other immunocompromised status, require special attention and suspicion, even when there is no history of tuberculosis exposure or evidence of thoracic active disease [7].

In our case, the tuberculous psoas abscess was masked by initial pus culture growth of MSSA, which contributed to a longer delay in treatment and serious complications. Antibiotic therapy for the staphylococcal infection had improved the septic conditions but did not block the progression of the spine involvement. After all, the psoas muscle infection was also originating from unknown Pott’s disease, thus leading to the conclusion of MSSA and MTB unusual coinfection.

These cases must be investigated with pus samples for mycobacterial culture as soon as possible. Isolation of other bacteria from an infected site does not mean the exclusion of a simultaneous tuberculosis infection.

Early recognition and targeted treatment are the principal means of ensuring tuberculosis control, preventing irreversible complications.

## Conclusions

Although* Staphylococcus aureus* is the major cause of psoas abscess, in the absence of clinical response, it is also important to consider and exclude *Mycobacterium tuberculosis*. This clinical case intends to grow awareness among the medical community, thus providing the highest quality of care to our patients.
